# Machine-Learning Approach for Forecasting Steam-Assisted Gravity-Drainage Performance in the Presence of Noncondensable Gases

**DOI:** 10.1021/acsomega.2c01939

**Published:** 2022-06-07

**Authors:** Serhat Canbolat, Emre Artun

**Affiliations:** † Department of Petroleum and Natural Gas Engineering, Near East University, TRNC, Mersin 10, Nicosia, Turkey 99138; ‡ Department of Petroleum and Natural Gas Engineering, Istanbul Technical University, Maslak, Istanbul, Turkey 34469

## Abstract

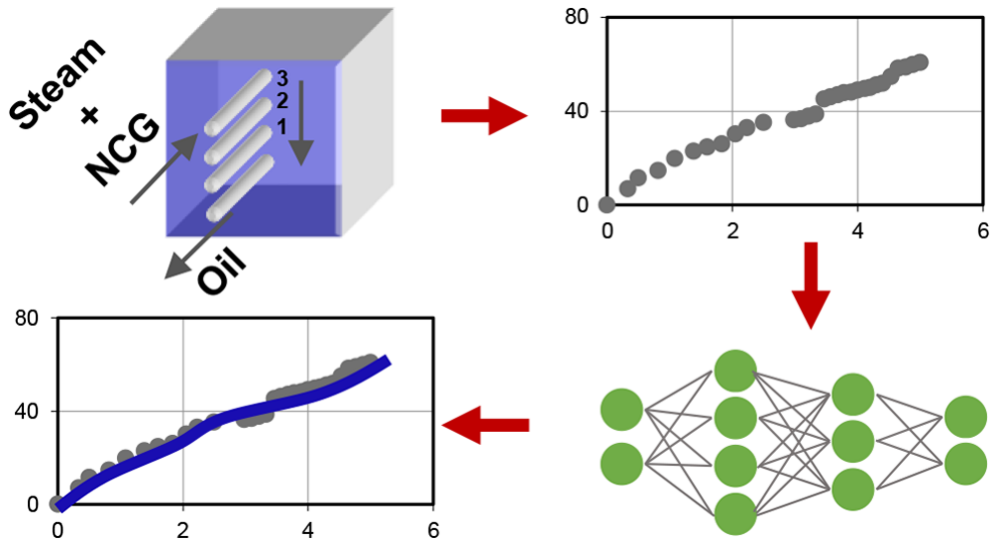

Steam-assisted gravity drainage (SAGD) is an effective enhanced oil recovery method for heavy oil reservoirs. The addition of certain amounts of noncondensable gases (NCG) may reduce the steam consumption, yet this requires new design-related decisions to be made. In this study, we aimed to develop a machine-learning-based forecasting model that can help in the design of SAGD applications with NCG. Experiments with or without carbon dioxide (CO_2_) or *n*-butane (*n*-C_4_H_10_) mixed with steam were performed in a scaled physical model to explore SAGD mechanisms. The model was filled with crushed limestone that was premixed with heavy oil of 12.4° API gravity. Throughout the experiments, temperature, pressure, and production were continuously monitored. The experimental results were used to train neural-network models that can predict oil recovery (%) and cumulative steam–oil ratio (CSOR). The input parameters included injected gas composition, prior saturation with CO_2_ or *n*-C_4_H_10_, separation between wells, and pore volume injected. Among different neural-network architectures tested, a 3-hidden-layer structure with 40, 30, and 20 neurons was chosen as the forecasting model. The model was able to predict oil recovery and CSOR with *R*^2^ values of 0.98 and 0.95, respectively. Variable importance analysis indicated that pore volume injected, distance between wells, and prior CO_2_ saturation are the most critical parameters that would affect the performance, in agreement with the experiments.

## Introduction

**Figure 1:**
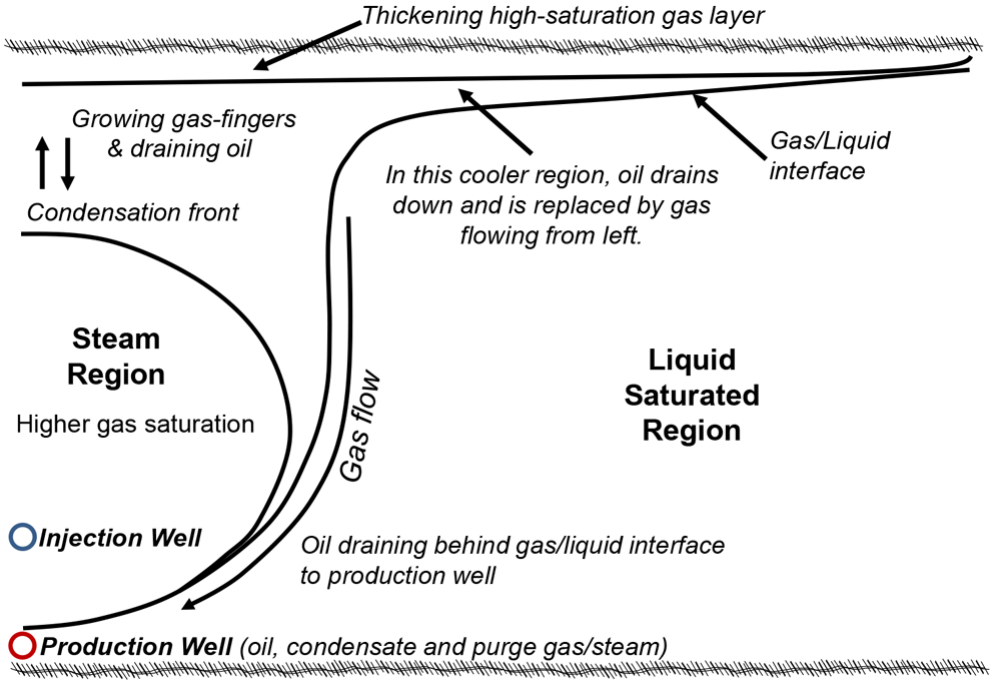
Schematic illustration of the SAGP mechanism.

Steam-assisted gravity drainage (SAGD) is an effective enhanced oil recovery (EOR) method for heavy-oil reservoirs. The process includes two horizontal wells.^1^ Steam is pumped from the upper well into the reservoir, and when heated, heavy oil is mobilized under gravity to the producer located below. A steam chamber forms due to the accumulation of steam within the pay zone. The level of heavy oil heating near the chamber’s edge is a significant factor in the efficiency of this approach. The greater the heated heavy oil layer at the steam chamber’s edge, the greater is the thickness of the movable oil at the reservoir’s bottom and the lower is the viscosity of the heavy oil in the movable layer.^2^ The need for high volumes of steam, particularly in thin, low-quality reservoirs, is a significant restriction of SAGD due to the requirement of continuous steam production on the surface. This could be avoided by adding noncondensable gases (NCG) such as carbon dioxide (CO_2_) or methane (CH_4_) (i.e., the steam and gas push (SAGP) mechanism) or additionally by injecting liquefied petroleum gases such as propane (C_3_H_8_) or *n*-butane (*n*-C_4_H_10_) (i.e., the vapor extraction (VAPEX) process).^3,4^ In steam and gas push (Figure 1), NCG such as carbon dioxide (CO_2_), methane (CH_4_), and nitrogen (N_2_) are coinjected with steam. Because NCG have a lower density than condensate steam, they begin to rise within the reservoir and accumulate at the top of the depletion chamber.^2^

2D experiments to evaluate the performance of the SAGD process in the presence of NCG demonstrated promising results in terms of reducing the residual oil saturation.^5^ The process appeared to be dominated by gas fingers. Reduced steam-chamber growth and oil rates were observed in the presence of NCG in other experiments.^4^ However, comparable oil recoveries were observed with and without NCG in core plug experiments. Other sets of experiments to evaluate the wind-down process by injecting NCG after SAGD resulted in a recovery of 12.5%.^6^ The temperature profile demonstrated that the hot chamber continued growing when the steam injection stopped due to the expansion/pressurization with NCG addition. In another study, two sets of experiments were conducted to evaluate the NCG distribution in the SAGD chamber when there was initial gas saturation: one at low pressure and one at high pressure.^7^ For the low-pressure case, free noncondensable gas tended to accumulate at the steam front of a steam chamber. However, there was also lower liquid saturation at the top of the cell, most likely due to gas accumulation. For the high-pressure test at early times, the density and temperature profiles evolved together. At later times, the gas phase expanded more than the chamber described by the temperature profile, indicating the presence of gas at the top of the pack. At the end of the experiment, the cell was opened, and the top of the model, although depleted, tended to be darker, indicating that this region never reached full steam temperature.

In another study, the performance of SAGD with initial free gas saturation was evaluated with two experiments.^8^ It was argued that gas liberated during the cold oil primary production could negatively affect the SAGD process. The gas saturation delayed the heat transfer to the bitumen and dramatically retarded the growth of the chamber. The high intensity of gas at the top created a cushioning effect that reduced heat loss to the overburden, and the gas at the top forced oil down, assisting in improved drain gravity to the lower producer. Because of the presence of NCG and the lower partial pressure of steam, the temperature was lower, and thus heat transmission to the heavy oil was reduced. The latter contributed to lower levels of oil mobilization and production. The convected condensate added energy to the liquid, but as the liquid concentration decreased when the condensate filled the reservoir, so did the oil’s relative permeability. This also accounted for the increased relative permeability to gas compared to SAGD. The oil saturation showed a depleted oil zone. However, the relative permeability of the oil was low compared to SAGD, implying that, although the oil was mobilized, the relative permeability effects that arose from the increased gas concentration in the system limited the flow of the oil. In other words, because of the high gas concentration, the relative permeability of the oil to the rock was reduced. This adversely affected the oil-phase mobility, which led to reduced oil drainage. The water saturation profile was high for the SAGD case because more steam was injected into the system. This increased the water saturation coming from the steam condensate, hence resulting in the higher relative permeability to water. As a result of the lowered oil viscosity, the oil mobility diminished due to lower temperatures at the chamber’s edge.^9^ Co-injection of NCG with steam was believed to provide several benefits in the SAGD process, such as higher energy efficiency and the ability to maintain pressure in the reservoir. The injected NCG tended to accumulate in the steam chamber, and gas fingering penetrated the reservoir ahead of the steam front due to the higher relative permeability.^10^ The gas breaching was more complex than can be related only by the interaction between gas/steam that was mainly controlled by partial pressure. The gas fingering made the system more stable and considered such stabilization mostly at higher temperatures, a factor defined as a gas mobility factor.

SAGD is a multiphysical process involving simultaneous heat and mass transmission.^11^ As a result, empirical and analytical methods are not able to effectively predict SAGD performance. As long as a sufficient amount of good-quality data are available, numerical reservoir simulation, as a robust physics-based tool, has been an effective approach for predicting the performance of the SAGD process over its entire life cycle.^12^ However, as the numerical reservoir model becomes more complex, the resources that are necessary become considerably high. As a result, computationally more efficient data-driven models become valuable and more practical. This is an effort to design a procedure that may use the provided data sets to build a data-driven model and provide accurate forecasts while using less computational and storage resources.^13^ Machine-learning algorithms are used to design and develop such applications to extract complex relationships between system and performance characteristics. In addition to their applications for unconventional reservoirs^14−16^ and secondary recovery,^17−19^ successful EOR forecasting and screening models have also been developed. Evaluations of different EOR methods,^20^ chemical flooding methods,^21−24^ cyclic pressure pulsing,^25,26^ and thermal methods^27−30^ including SAGD^31,32^ were made using data-driven models. A combination of models for different EOR processes can result in an comprehensive toolbox that would help to optimize the design of potential EOR applications.^33^

Neural networks were employed to develop forecasting models for SAGD. Proxy models that used numerical-simulation results for model training helped to reduce the computational load.^31,32^ Data collected from the literature were used to train a neural network that can forecast recovery in SAGD operations.^34^ By combining reinforcement learning with optimization algorithms and a numerical simulator, steam injection in SAGD was optimized.^35,36^ Machine learning was also employed to efficiently optimize steam allocation in a multipad SAGD reservoir model of Athabasca formation.^37^ Numerical models based on data gathered from several Athabasca oil sands were used for dynamic data integration for shale-barrier characterization via convolutional neural networks.^38^ Seismic data were combined with operational data from wells to forecast dynamic changes observed in 4D seismic during SAGD.^39^ Physics-informed machine-learning models have also been an active area of development in the machine-learning community. These models target reduced computational load and improved accuracy. This idea was also demonstrated for a SAGD example with successful results.^40^

The aforementioned examples highlight different uses of machine-learning algorithms to either forecast SAGD performance or optimize SAGD operations. Because of the difficulty in accessing real data, most studies consider numerical-simulation results to develop data-driven models. Numerical simulators are useful to generate significant amounts of related data. However, the true value of machine-learning algorithms comes into the picture when real observations are used for model training. This is more evident in the absence of a reliable numerical or analytical model. The primary objective of this study is to use experimental results in the development of a machine-learning-based forecasting model for the optimized design of SAGD applications with NCG. The experiments helped to demonstrate how the addition of particular amounts of NCG contributes to the effectiveness of a SAGD application. By using the forecasting model, steam use can be minimized while introducing new design considerations.

## Methodology

### Experimental Study and Data Collection

**Figure 2:**
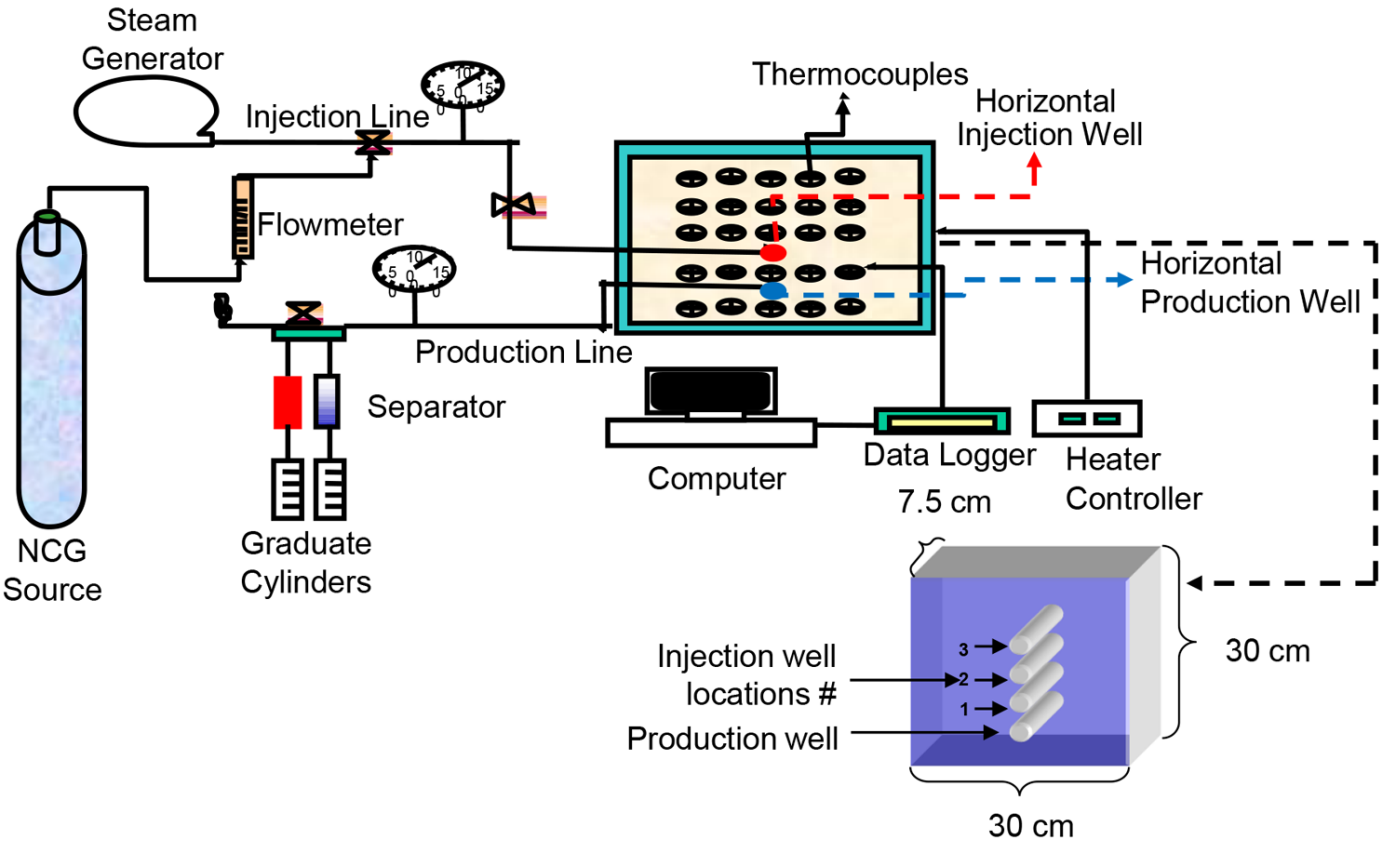
Experimental setup of SAGD experiments with noncondensable gases.

The physical scaled model setup, as shown in (Figure 2), consisted of three primary components: injection equipment, physical model, and production facilities. A steam generator and a 10 MPa pressured CO_2_/*n*-C_4_H_10_ bottle were employed on the injection side. CO_2_ or *n*-C_4_H_10_ was metered into the live steam using a flow meter. A resistance heater connected to a temperature controller was used to keep the injection line at the same temperature as the steam generator. This ensured that superheated steam was delivered into the physical model at a pressure of 280 kPa and a temperature of 140 °C. The injection well was built using perforated tubing wrapped in a wire screen. The screen was implemented to avoid the unconsolidated porous media from migrating.

**Figure 3:**
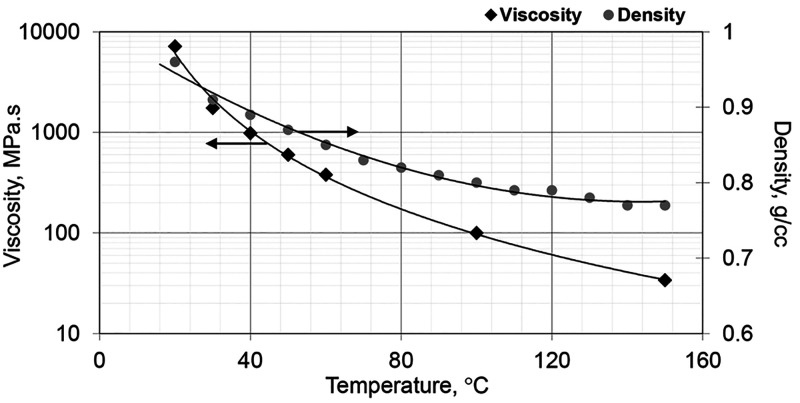
Temperature-induced changes in crude oil viscosity and density.

The model was 30 cm tall, 30 cm wide, and 7.5 cm thick, and it was made of stainless steel. The model was preheated to 50 °C using a thermal blanket. The injection well was located 5 cm above the production well. Figure 2 shows three arrays of 25 thermocouples spaced by a distance of 2 cm positioned within the width of the model. Thermocouples were inserted into the model through welded-on tube fittings and connected to a computer. This arrangement allowed for constant 3D temperature monitoring of the model. A pressure gauge and two fluid separators made up the production system. The production well was similarly made of perforated tubing wrapped in a wire screen to prevent the porous material from migrating. It was heated to avoid clogging with cooled viscous oil. Production was measured in volumetric units. To meet the requirements of a water-wet system, crushed limestone (mesh size 0.5–1.4 mm) was washed with deionized water. The limestone/water slurry was mixed with crude oil, resulting in oil and water saturations of 75% and 25%, respectively. Initial saturations were kept constant throughout the sessions. Table 1 shows the rock and fluid properties of the model. The oil sample was a viscous crude oil with a gravity of 12.4° API extracted from the Bati Raman field. Figure 3 depicts the temperature dependence of viscosity and density for this oil. The viscosity was ∼600 cP at the initial experimental temperature of 50 °C. Injection and production pressures were measured, as well as the characteristics of the produced oil and water.

**Table 1:** Properties of the Experimental Model

crude oil	Bati Raman
API gravity (°API)	12.4
porosity (%)	38
permeability (darcy)	5
pore volume (cm^3^)	2700
water volume (cm^3^) and saturation	675–25%
oil volume (cm^3^) and saturation	2025–75%
mass of sand (g)	10565
matrix (0.5–1.4 mm)	crushed limestone
sand density (g/cm^3^)	2.5

The model was vertically positioned and preheated to 50 °C. The steam generator was turned on and set to 280 kPa and 140 °C. If NCG were to be utilized, they were mixed with the steam after the temperature and pressure were reached for injection. The steam and gas mixture was then fed into the model in a 1:1.29 ratio. To avoid condensation, the supply line was kept above 100 °C. Throughout the studies, the injection line temperature, temperature profile in the model, pressure, and production data were continually collected. When the steam–oil ratio began to rapidly grow, the experiment was called off.

The dimensional similarity between the model and the field conditions can be obtained for the proposed theory by scaling the dimensionless time, *t*′:^1^
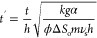
1where *t* is time (min); *h* is the reservoir height in (m); *k* is the effective permeability (m^2^); *g* is the acceleration due to gravity (m/s^2^); α is the thermal diffusivity (m^2^/day), ϕ is the porosity (fraction); Δ*S*_o_ is the difference between the initial and residual oil saturation; *m* is the crude oil viscosity parameter, which a function of the viscosity–temperature characteristics of the oil, the steam temperature, and the reservoir temperature (dimensionless); and ν_s_ is the kinematic viscosity of the oil at the temperature of the steam (m^2^). As a result, dimensional similarity was provided by a dimensionless number, *B*_3_:
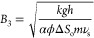
2

### Development of the Machine-Learning-Based Forecasting Model

Using the experimental results, a machine-learning-based forecasting model was developed using neural networks. An artificial neural network is a mathematical and rather simplified representation of a biological neural network that can extract patterns from multidimensional data as well as nonlinear relationships between variables. Neural networks form the backbone of deep learning algorithms that are used in many artificial intelligence applications. In this study, the experimental design parameters such as injected gas composition, prior presence of CO_2_ or *n*-C_4_H_10_, separation between wells, and pore volume injected were used as input parameters. Binary categorical variables can be converted into continuous variables using the dummy variable approach.^41^ Categorical descriptions of the prior presence of noncondensable gases were encoded into continuous variables (CO2P for CO_2_ and ButaneP for *n*-C_4_H_10_) using the following convention:
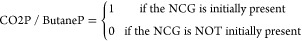
3The injected gas composition was inputted using the following variables: Steam, CO2, and Butane for the concentrations of steam, CO_2_, and *n*-C_4_H_10_, respectively. Other inputs were *x* for the separation between injection and production and PVinj for the pore volume injected. The model was designed to forecast oil recovery (%) and cumulative steam–oil ratio as the performance characteristics. The neuralnet library^42^ in the R environment^43^ was used to design, train, and evaluate the neural-network models for this purpose. Eighty percent of the data set of 9 experiments was used for training (180 data points), and the remaining 20% (45 data points) was used for testing. All variables were normalized between 0 and 1 before being used in training. A resilient backpropagation algorithm with weight backtracking^44^ was used with logistic activation functions in hidden layers and a linear function in the output layer. The sum of squared errors was used as the loss function with a stopping threshold of 0.01 for the partial derivatives of the loss function. Another stopping criterion was the maximum number of iterations, which was defined as 1 × 10^5^.

Several neural-network architectures were tested and evaluated. To evaluate the predictive capability of the neural network, two performance measures were used. Root-mean-square error (RMSE) measures the square root of the average squared difference between real values and their associated predictions,^45^
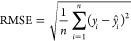
4where *n* is the number of training patterns, *y*_*i*_ is the real value of the output variable (oil recovery and cumulative steam–oil ratio), and *ŷ*_*i*_ is the predicted value of the output variable for training pattern *i*. The *R*^2^ statistic (coefficient of determination) is another way to assess the quality of the fit, representing the proportion of the variance explained in the forecasting model (the neural network),^41^
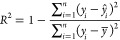
5where *y̅* is the mean of the output variable.

For the selected model, the importance of variables was evaluated using the connection-weight approach, which takes into account the optimized weight values in the connection link.^46,47^ This approach calculates variable importance by considering the product of the raw input–hidden and hidden–output connection weights between each input and output neuron. Finally, the product is summed across all hidden neurons. It is important to note that this method is only useful to compare variable importance within a single model. It is not recommended to make comparisons of the importance between different models.^48^

## Results and Discussion

### Experimental Results

**Figure 4:**
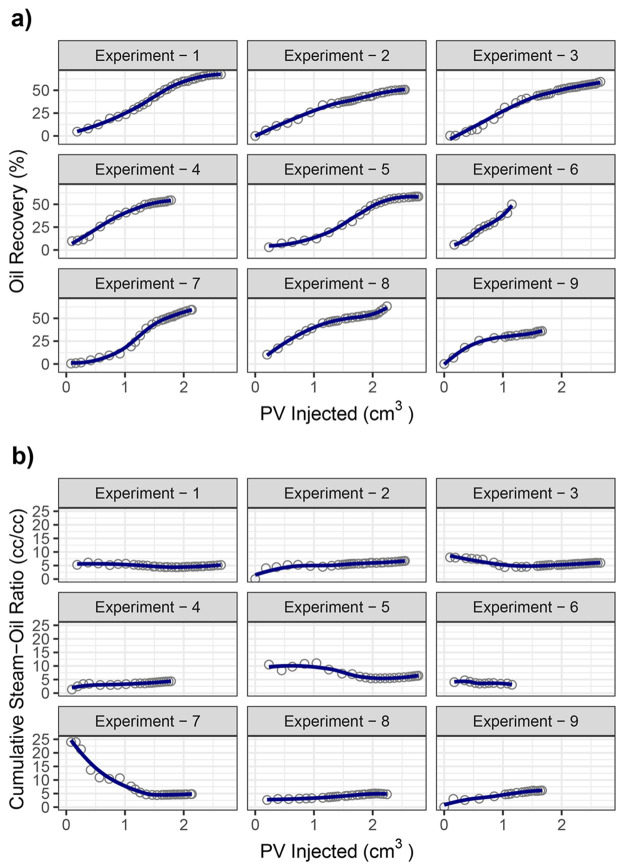
(a) Oil recovery and (b) cumulative steam–oil ratio as a function of pore volumes injected for all nine experiments (points indicate real experimental data, and lines indicate locally weighted smoothing curves).

**Figure 5:**
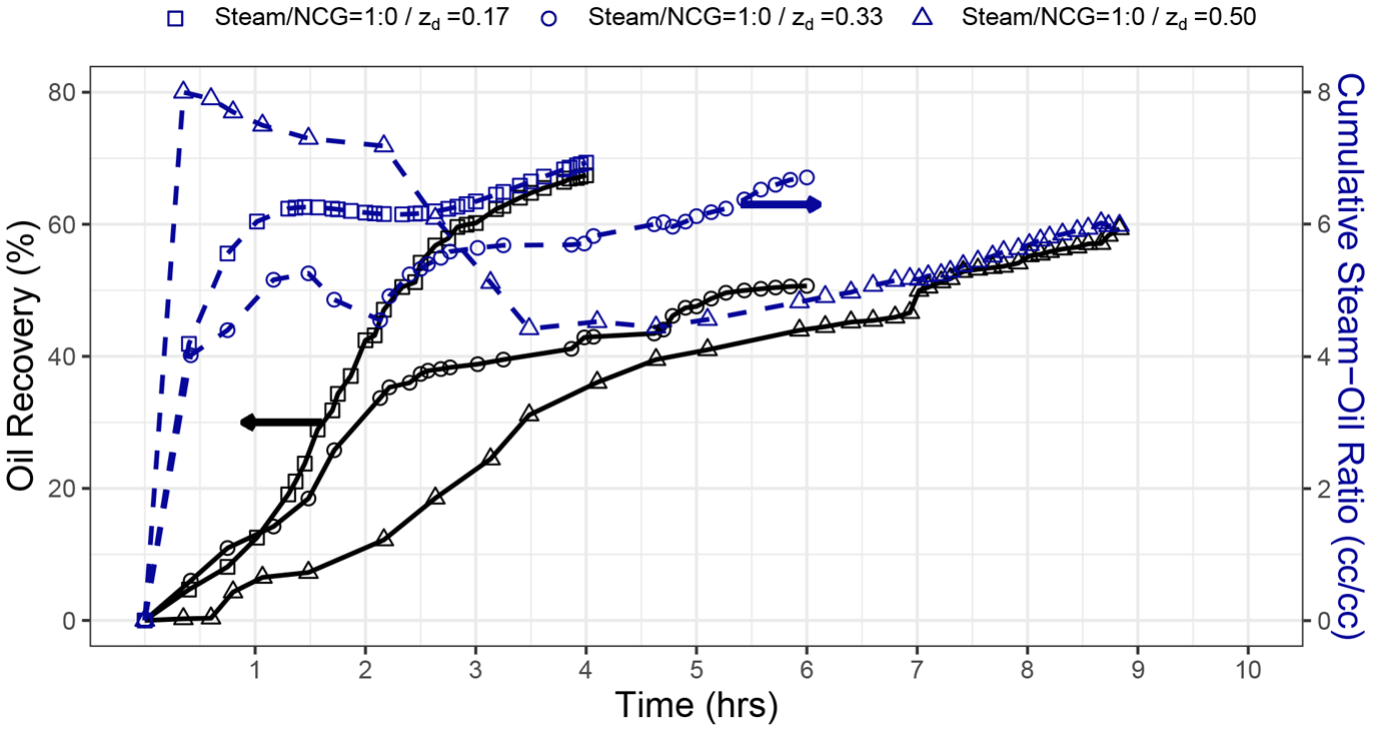
Comparison of SAGD experiments at different producer–injector spacing for *z*_d_ = 0.17, *z*_d_ = 0.33, and *z*_d_ = 0.50.

A summary of all experiments and key results is shown in Table 2. The producer–injector spacing is characterized by a dimensionless number (*z*_d_) that is the ratio of the well spacing to the reservoir model’s height. The heated area as a function of time was the highest for the smallest well separation. An overview of oil recovery and cumulative steam–oil ratio as a function of pore volumes injected for all experiments can be seen in Figure 4. Locally weighted smoothing was also added to better visualize the trends. The results of conventional SAGD experiments are summarized in Figure 5. Steam volumes were calculated in terms of cold-water equivalent (cwe). The shortest well spacing (*z*_d_ = 0.17) resulted in the highest, most rapid overall production, which is consistent with the observation of optimum early time heating with small well separations. Toward the end of the experiment, all of the production curves indicated gravity drainage of the oil.

**Table 2:** Summary Results of the Experiments for SAGD with NCG Co-injection

no.	type	*z*_d_	*t*, min	% oil rec.^a^, OOIP	vol ratio, gas/steam	CSOR^a^ cwe, cc/cc	avg pres., psi	avg. inj. temp., °C	flow rate, cc/min
1	SAGD	0.17	240	67.4		6.93	46.0	139	36.4
2	SAGD	0.33	360	50.7		6.71	50.7	134	47.3
3	SAGD	0.50	530	59.3		5.98	47.0	144	39.0
4	SAGD (with CO_2_initially present)	0.17	300	54.5		4.36	50.0	139	46.0
5	SAGD (with *n*-C_4_H_10_ initially present)	0.17	215	58.5		6.41	37.0	144	22.0
6	SAGD-CO_2_	0.33	440	63.2	4.41	3.08	46.0	143	47.3
7	SAGD-CO_2_	0.17	300	60.7	1.29	4.8	38.0	140	24.0
8	SAGD-CO_2_	0.17	566	49.9	4.41	4.73	50.0	140	36.4
9	SAGD-*n*-C_4_H_10_	0.17	300	36.2	1.29	6.14	37.0	141	22.0

a End-of-the-experiment values.

#### Steam/CO_2_ Experiments

Studies were carried out to evaluate the effect of NCG on the heating and oil recovery of SAGD. Simultaneously with steam, CO_2_ or *n*-C_4_H_10_ was introduced. Two flow rates were used: one comparable to the SAGD experiments and one higher. The impact of well spacing on the production efficiency was also considered for the CO_2_-injection trials by modifying the location of the injection well. The influence of CO_2_ was investigated for an identical CO_2_/steam ratio (4.41) at two different well separations (*z*_d_ = 0.17 and *z*_d_ = 0.33).

**Figure 6:**
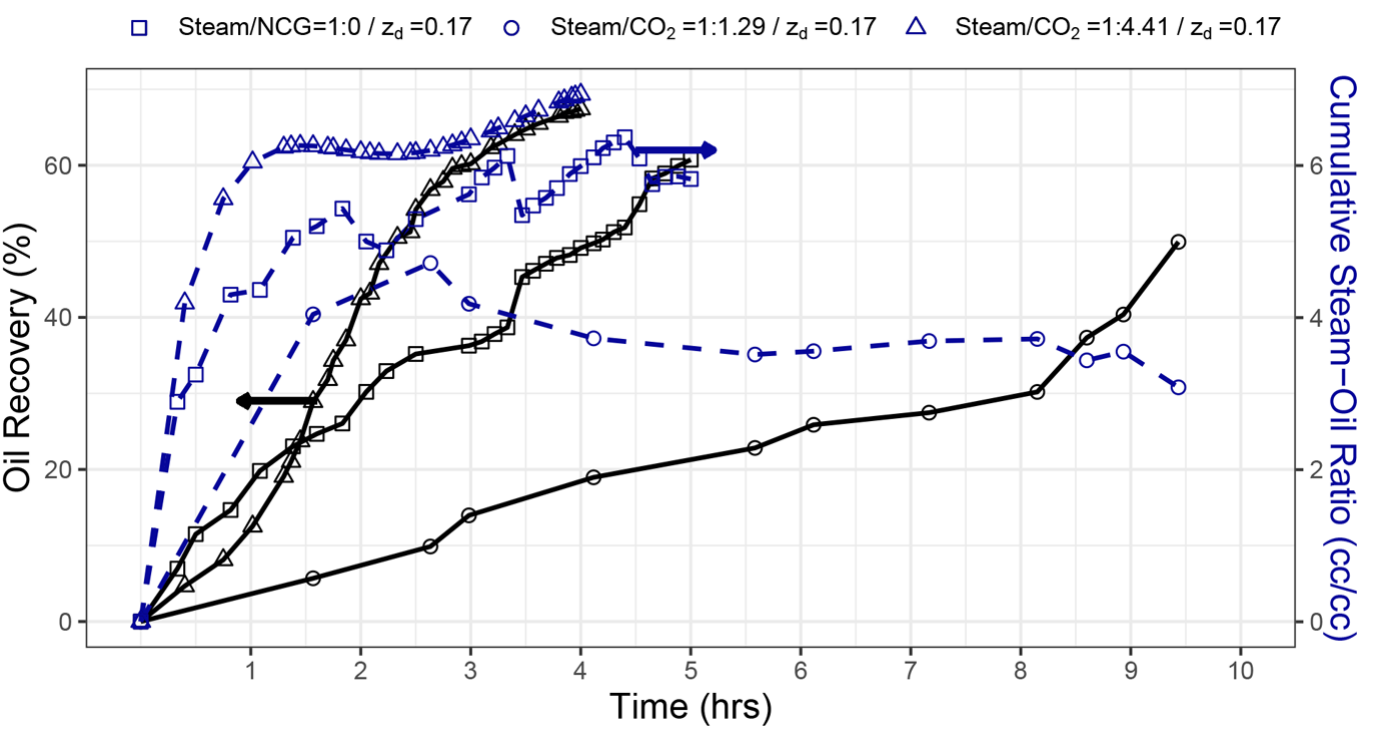
Comparison of SAGD with and without NCG experiments at different volumetric ratios for *z*_d_ = 0.17.

The model was chilly during the earliest stages of injection, and the majority of the injected steam condensed. The NCG rose in the form of fingers, which displaced very little oil. Heating was limited in scale due to the low heat capacity of the gas. As a thin gas zone, this gas accumulated under the model’s top impermeable border. In this respect, the NCG did actually carry pressure to the reservoir’s upper sections. The rising gas introduced temperature and pressure gradients into the chamber. The addition of carbon dioxide did not boost the eventual oil recovery, as indicated in Figure 6, but it did extend the heating period and project life. The steam–oil ratios were lower than in standard SAGD studies. In the same way as SAGD performed better in terms of heating and oil recovery, the smaller well separation performed better in terms of heating and oil recovery.

**Figure 7:**
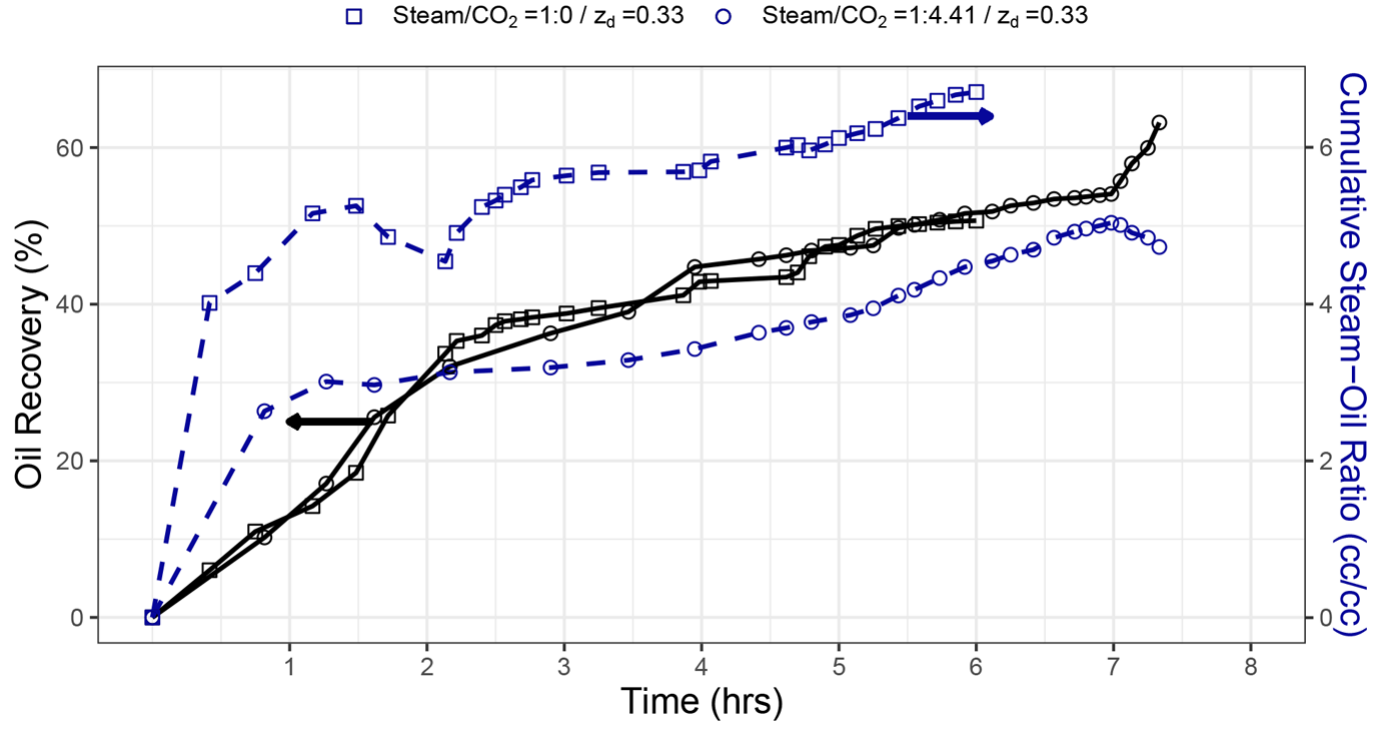
Comparison of SAGD with and without NCG experiments at different volumetric ratios for *z*_d_ = 0.33.

Several experiments were carried out by varying the steam–CO_2_ ratio from 0 to 4.41 while using the same injection–production location at *z*_d_ = 0.33. The time required to heat the system increased as the CO_2_ concentration in the injected mixture increased due to a decrease in latent and sensible heat per unit of injectant. The oil recovery and recovery rates dropped (Figure 7), but the decline in the steam–oil ratio became more pronounced as the amount of gas in the combination increased.

#### Steam/*n*-C_4_H_10_ Experiments

**Figure 8:**
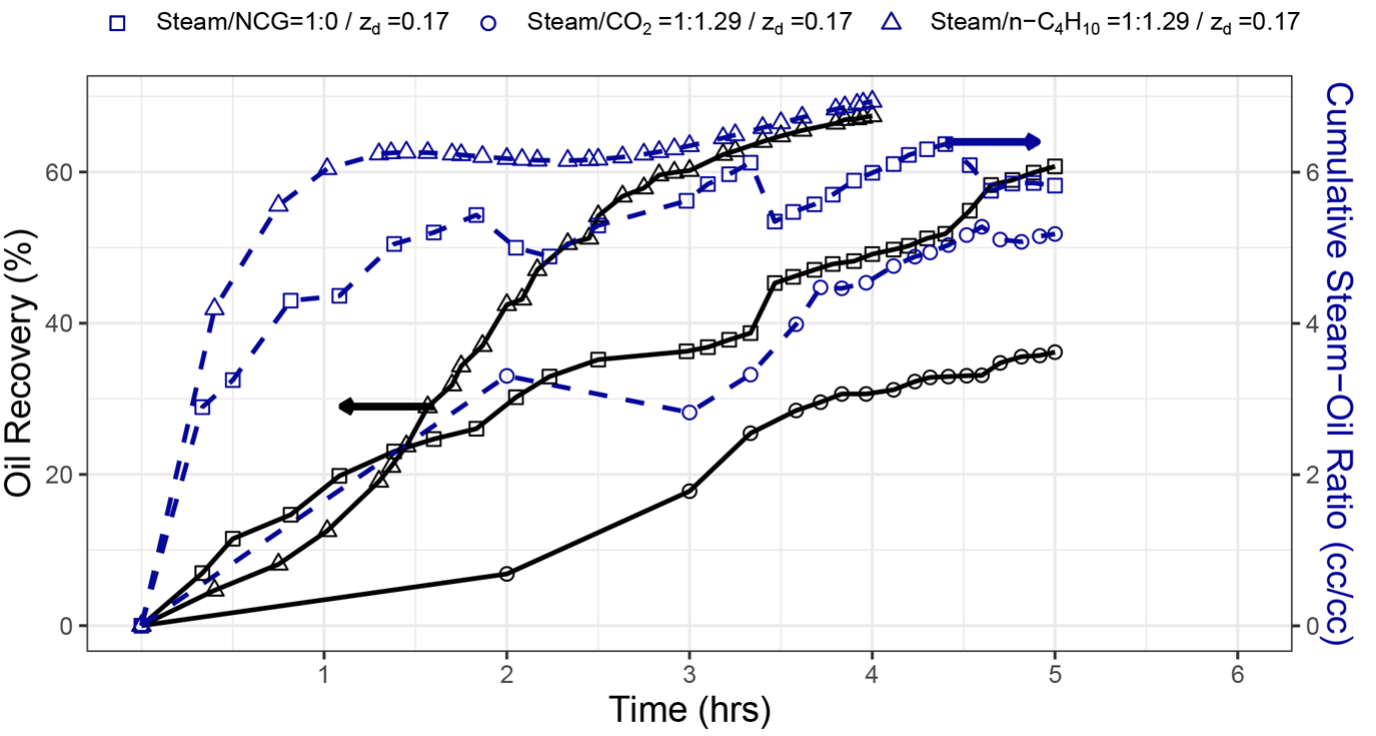
Comparison of SAGD with and without NCG experiments with the same volumetric ratio for *z*_d_ = 0.17.

Adding a hydrocarbon gas to the steam was also considered. A steam–*n*-butane combination was injected at the same concentration as in the steam–CO_2_ trials and at the same injection–production locations (*z*_d_ = 0.17). Oil recoveries and steam–oil ratios (Figure 8) were lower, as expected, as compared to traditional SAGD recovery. CO_2_ surpassed *n*-C_4_H_10_ in terms of performance.

#### Effects of the Initial Presence of NCG

**Figure 9:**
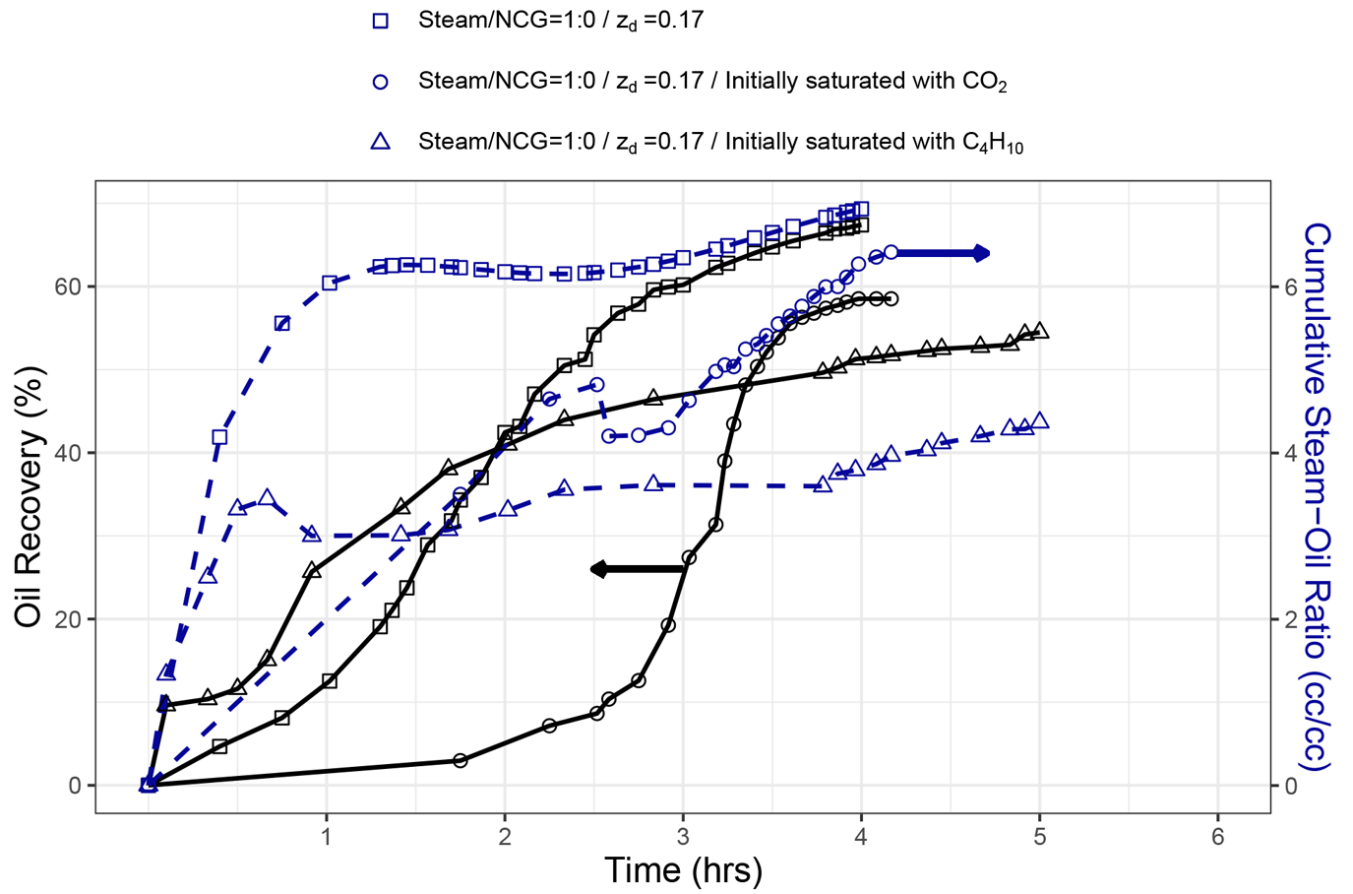
Comparison of SAGD with and without initial NCG saturation experiments for *z*_d_ = 0.17.

Finally, the existence of initial gases in the system was studied at the injection–production location for *z*_d_ = 0.17. The main point of interest here was the possibility of steam injection following primary production via solution gas drive or immiscible, isothermal gas injection. To achieve initial nonzero gas saturation, several pore volumes of CO_2_ or *n*-C_4_H_10_ were introduced, and the system was then turned off for 24 h. Small amounts of oil were produced during injection. By mass balance, the average initial gas saturations for CO_2_ and *n*-C_4_H_10_ were thus 8.35% and 3.0% of the original oil in place, respectively. Then, conventional SAGD was initiated. Figure 9 depicts the observed oil recovery and steam–oil ratios during the experiments. Initial saturation of *n*-butane had a positive effect on oil recovery when compared to simultaneous gas and steam injection. This was most likely due to the heavy oil’s viscosity being reduced as a result of *n*-C_4_H_10_ dissolving in the oil. The first gas saturation most likely generated low resistance paths for the steam to travel through the viscous oil that was saturating the model.

The SAGD experiment was carried out with the values reported and used for the settings given in Table 3 for the Bati Raman field. Table 4 shows the scaling results for the experiments. For the conventional SAGD, the determined dimensional similarity suggested that 1 min of experiment time corresponded to 18.78 days in the field.

**Table 3:** Standard Values for Scaling SAGD

parameter	laboratory	field
α (m^2^/s)	8.10 × 10^–7^	8.10 × 10^–7^
ν (m^2^/s)	5.75 × 10^–6^	7.00 × 10^–5^
Δ*S*_o_	0.5025	0.5025
ϕ	0.38	0.25
*k* (m^2^)	2.48 × 10^–13^	1.19 × 10^–14^
*g* (m/s^2^)	9.81	9.81
*w* (m)	0.075	100
*h* (m)	0.225	37

**Table 4:** Calculated Parameters for Scaling SAGD

parameter	laboratory	field
*m*	4	4
*kg* (m^3^/day^2^)	0.018	0.001
*h* (m)	0.225	37
ϕΔ*S*_o_	0.191	0.126
α (m^2^/day)	0.07	0.07
ν (m^2^/day)	0.497	6.048
*B*_3_	0.39	0.39
*t*′	120.3	0.0044
*t*	1 min	18.78 days

### Machine-Learning-Based Forecasting Model

Table 5 shows the model performance for different neural-network architectures of 1, 2, and 3 hidden layers with varying hidden neurons in each layer. The table shows the *R*^2^ and RMSE results obtained for both oil recovery and cumulative steam–oil ratio for their training and testing portions. While satisfactory results were obtained with all configurations, two architectures stood out with the highest accuracy results: (1) 1 hidden layer (40 neurons) and (2) 3 hidden layers (40–30–20 neurons). The 3-hidden-layers model resulted in lower RMSE for the testing set of the oil recovery. The input parameters included different dimensions of the problem such as static model properties and dynamic operational conditions as a function of time. As a result, the model with 3 hidden layers with 40, 30, and 20 neurons in each layer was selected to allow for sufficient complexity to handle multiple types of information presented as inputs and associated nonlinearities in the data. With *R*^2^ values >0.95, RMSE of 11.3% oil recovery, and 2.3 cc/cc cumulative steam–oil ratio, the model has a reasonable prediction accuracy.

**Table 5:** Training and Testing Performances of Different Neural-Network Architectures for the Prediction of Oil Recovery and Cumulative Steam–Oil Ratio

		training				testing			
		oil rec. (%)		CSOR		oil rec. (%)		CSOR	
no. of hidden layers	no. of neurons	*R*^2^	RMSE	*R*^2^	RMSE	*R*^2^	RMSE	*R*^2^	RMSE
1	10	0.99	26.0	0.97	7.1	0.98	14.9	0.90	3.2
	20	0.99	18.6	0.97	6.3	0.99	13.0	0.93	2.7
	30	0.99	27.1	0.96	8.1	0.99	13.6	0.88	3.5
	40	0.99	20.3	0.98	5.1	0.99	13.8	0.95	2.3
2	20–10	0.99	17.8	0.98	6.2	0.99	13.8	0.92	2.9
	30–20	1.00	16.5	0.97	6.7	0.99	13.9	0.91	3.1
	40–25	0.99	26.1	0.97	6.4	0.98	16.8	0.89	3.3
	50–30	1.00	15.5	0.99	4.6	0.99	11.1	0.93	2.7
3	15–10–5	1.00	15.4	0.99	4.4	0.99	10.5	0.94	2.4
	30–20–10	0.99	17.9	0.98	5.3	0.99	11.4	0.94	2.5
	40–30–20^a^	1.00	16.9	0.98	5.2	0.99	11.2	0.95	2.3
	50–35–20	1.00	14.9	0.99	4.0	0.99	9.9	0.93	2.6

a Selected model.

**Figure 10:**
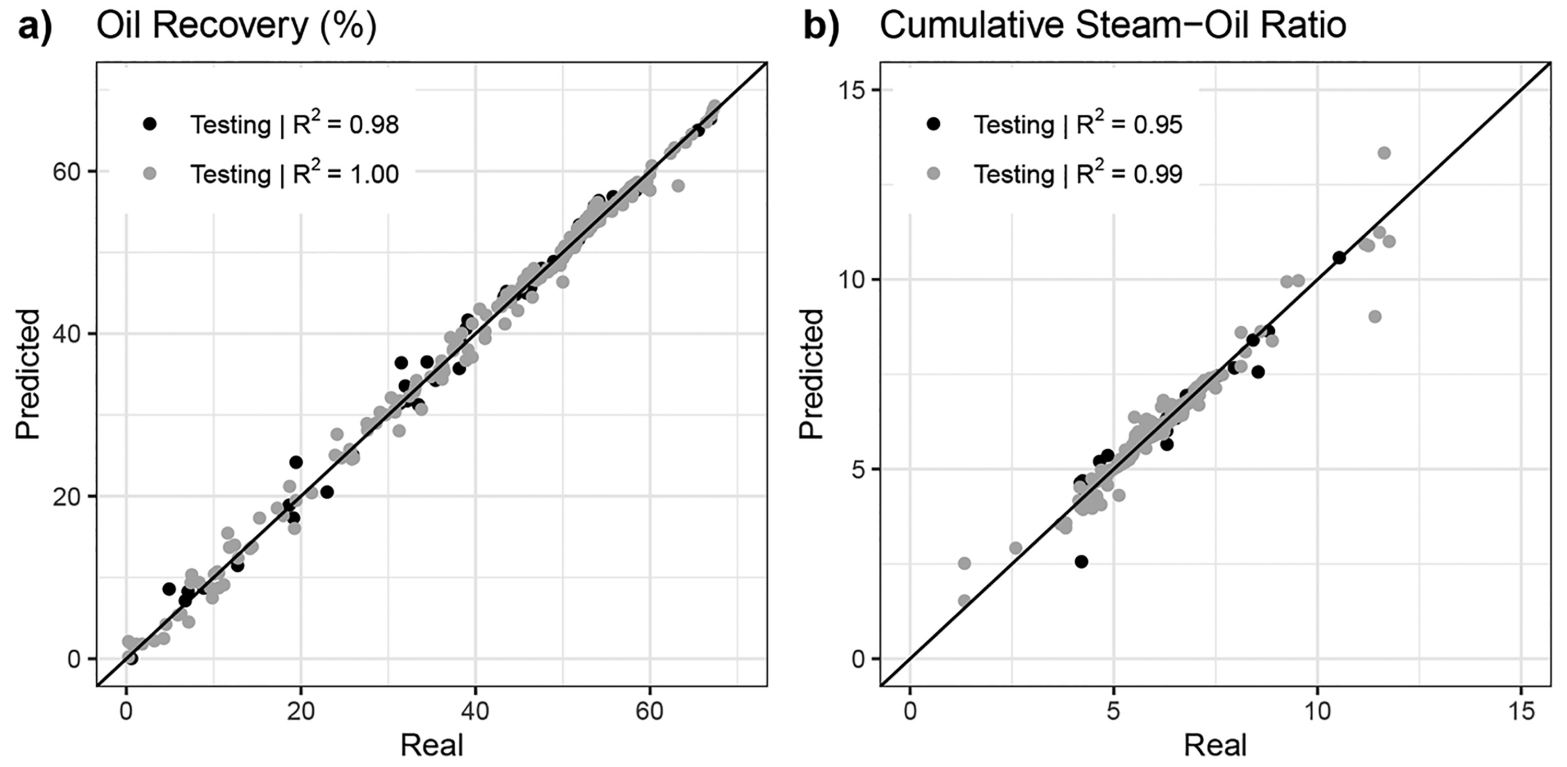
Real versus predicted crossplot for the training and testing sets of (a) oil recovery and (b) cumulative steam–oil ratio using the neural network with 3 hidden layers with 40, 30, and 20 neurons in each layer.

**Figure 11:**
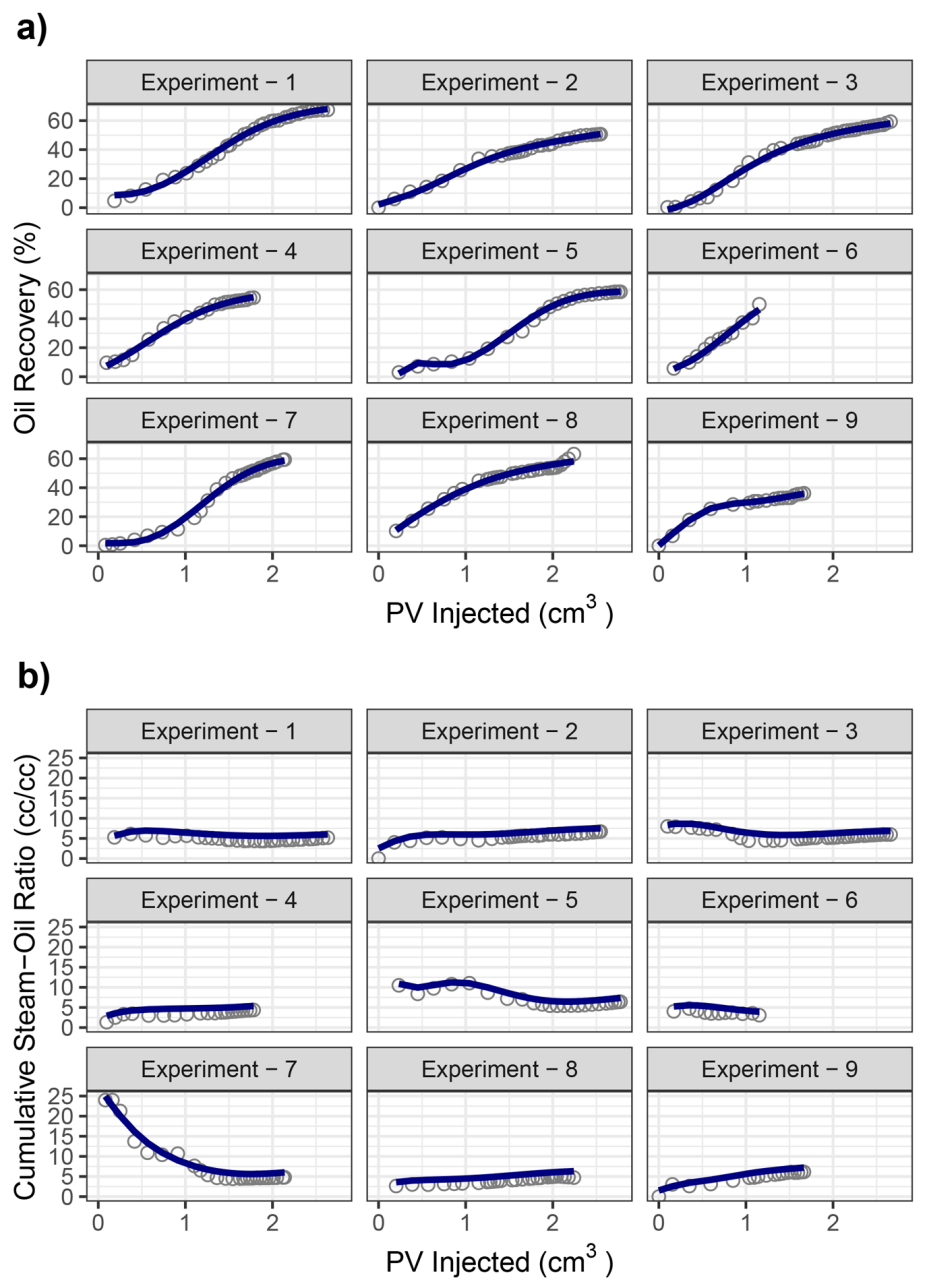
(a) Oil recovery and (b) cumulative steam–oil ratio as a function of pore volumes injected for all nine experiments (points indicate real experimental data, and lines indicate model predictions).

Figure 10 shows the cross-plots of real versus predicted values for both training and testing sets. As also seen in *R*^2^ and RMSE values, the accuracy in cumulative steam–oil ratio is somewhat lower than that in oil recovery. This is due to the definition of this variable as a ratio that includes two measured properties: cumulative oil and steam produced. To visualize the model’s predictive capability on the experimental data, Figure 4 is replotted by displaying the model predictions as a line on top of the experimental measurements. Figure 11 shows that the neural network is capable of forecasting the SAGD performance for different types of experiments with or without noncondensable gases.

**Figure 12:**
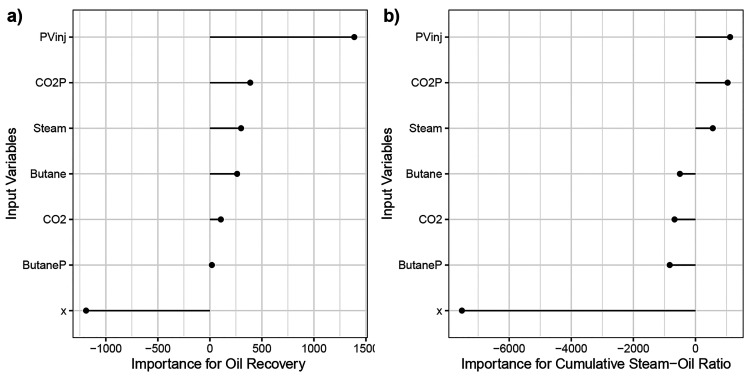
Importance of input variables for the prediction of (a) oil recovery and (b) cumulative steam–oil ratio using the connection-weight approach.

The variable importance plot obtained through the connection weight approach is shown in Figure 12. In this figure, it is possible to rank variables in terms of importance. Also, we can see whether an input variable positively or negatively affected the output variable. These plots indicate that pore volume injected is the most important variable positively affecting both oil recovery and cumulative steam–oil ratio. Distance between wells is also very critical, and it affects both output variables negatively. In terms of the injected gas composition, inclusion of CO_2_ and *n*-C_4_H_10_ reduces the steam–oil ratio, yet they contribute positively to oil recovery. The presence of CO_2_ and *n*-C_4_H_10_ positively contributes to oil recovery, which is potentially due to dissolution of these gases in oil and reduction in oil viscosity. These observations are in agreement with the experimental findings. This validates the reliability of the neural-network model in terms of capturing the fluid-flow dynamics of the SAGD process through experimental measurements.

**Figure 13:**
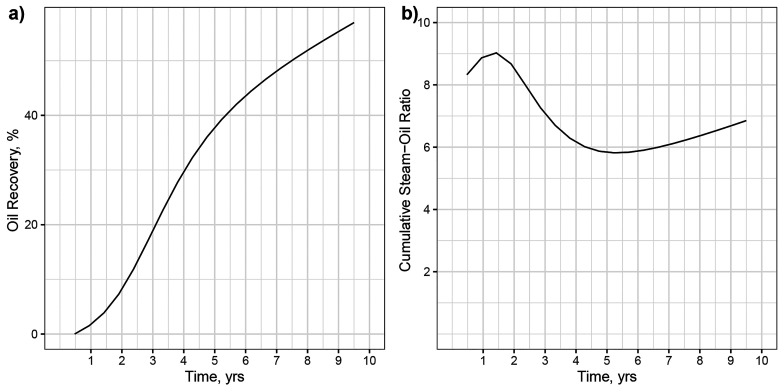
(a) Oil recovery and (b) cumulative steam–oil ratio vs field-scale time for Bati Raman application, forecasted using the neural-network model.

To demonstrate the practical application of the developed model, the case presented earlier for upscaling was considered. The parameters shown in Table 3 are input to the machine-learning model to forecast the oil recovery and cumulative steam–oil ratio for a SAGD application as a function of pore volume injected. By converting the lab-scale time to field-scale time, the performance of the SAGD application was forecasted for 10 years (Figure 13).

## Conclusions

This study led to the development of a procedure that can be applied to design a SAGD-EOR process with coinjection of NCG. Through this procedure, different design operational parameters such as NCG concentration and well separation can be optimized to maximize oil recovery and minimize CSOR. This can be coupled with an economic evaluation for the field-scale application. The following key conclusions are drawn from the results obtained from this study:

(1) In the case of conventional SAGD, lower injector-to-producer well separation resulted in faster heating, higher recovery efficiency, and higher steam–oil ratio.

(2) When a noncondensable gas was injected with steam, the gas fingered rapidly toward the top of the model and then displaced oil downward. The gas created a thin insulating layer, delaying the formation of a steam chamber. The addition of NCG to steam lowered the total heat capacity of the injected hot fluid. As a consequence, steam/NCG mixtures exhibited steam condensation effects faster than steam injection alone. This restricted the upward transfer of heat.

(3) With smaller well separation, the steam condensation temperature and the steam–oil ratio declined with the volume of CO_2_. The heavy oil became less mobile in the steam chamber due to the lower temperatures and viscous oil. As a result, the heating time increased while accumulated oil recovery and recovery rate decreased.

(4) The neural-network-based forecasting model resulted in accurate prediction of the performance of SAGD experiments. Through dimensional scaling, the forecasts can be converted into field-scale time and upscaled to the field dimensions for the recovery of heavy oil using SAGD wells.

(5) Analysis of the weights of the optimum neural-network configuration highlighted pore volume injected, well separation, and initial presence of NCG as the most important variables affecting SAGD performance. This observation was in agreement with the experimental results.

Potential future work may include the expansion of the presented model with a more comprehensive data set. The model can be coupled with an optimization routine and possibly with economic parameters to determine optimum operational conditions for a field under consideration.
